# Bis[4-amino-*N*-(pyrimidin-2-yl)benzene­sulfonamidato](2,2′-bipyridine)manganese(II)

**DOI:** 10.1107/S1600536810017794

**Published:** 2010-05-22

**Authors:** Tian-Jing He, Yan-Shu Tan, Yun-Qiong Gu, Zhen-Feng Chen, Hong Liang

**Affiliations:** a Key Laboratory for the Chemistry and Molecular Engineering of Medicinal Resources (Ministry of Education of China), School of Chemistry & Chemical Engineering, Guangxi Normal University, Guilin 541004, People’s Republic of China

## Abstract

The title compound, [Mn(C_10_H_9_N_4_O_2_S)_2_(C_10_H_8_N_2_)], contains a distorted octa­hedral [Mn(sdz)_2_(bpy)] (sdz is the sulfadiazine anion and bpy is 2,2′-bipyridine) complex mol­ecule. A three-dimensional network is generated by N—H⋯N, N—H⋯O and C—H⋯O hydrogen bonds from the sulfadiazine ligands.

## Related literature

For mono-ligand sulfadiazine–metal complexes, see: Yuan *et al.* (2001 ▶); Wang *et al.* (2005 ▶). For mixed-ligand sulfadiazine–metal complexes, see: Ajibade *et al.* (2006 ▶); Brown *et al.* (1987 ▶); Hossain *et al.* (2006 ▶); Wang *et al.* (2009 ▶, 2010 ▶). For 2,2′-bipyridine–Mn(II) complexes, see: Chen *et al.* (1995 ▶); Cheng *et al.* (2004 ▶).
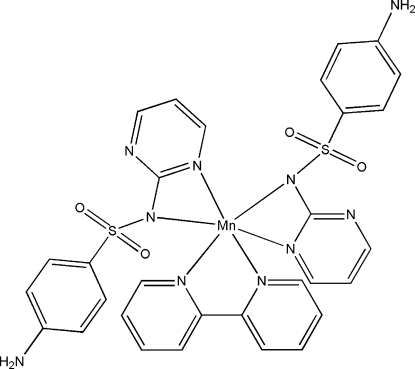


## Experimental

### 

#### Crystal data

[Mn(C_10_H_9_N_4_O_2_S)_2_(C_10_H_8_N_2_)]
*M* *_r_* = 709.67
Monoclinic, 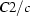

*a* = 20.121 (3) Å
*b* = 17.555 (3) Å
*c* = 17.956 (3) Å
β = 106.973 (4)°
*V* = 6066.4 (17) Å^3^
*Z* = 8
Mo *K*α radiation
μ = 0.63 mm^−1^
*T* = 193 K
0.25 × 0.17 × 0.15 mm

#### Data collection

Rigaku Mercury CCD diffractometer
Absorption correction: multi-scan (*REQAB*; Jacobson, 1998 ▶) *T*_min_ = 0.859, *T*_max_ = 0.912
28490 measured reflections
5533 independent reflections
4438 reflections with *I* > 2σ(*I*)
*R*_int_ = 0.077

#### Refinement

*R*[*F* ^2^ > 2σ(*F* ^2^)] = 0.073
*wR*(*F* ^2^) = 0.167
*S* = 1.17
5533 reflections
441 parameters
H atoms treated by a mixture of independent and constrained refinement
Δρ_max_ = 0.43 e Å^−3^
Δρ_min_ = −0.58 e Å^−3^

### 

Data collection: *CrystalClear* (Rigaku, 1999 ▶); cell refinement: *CrystalClear*; data reduction: *CrystalStructure* (Rigaku/MSC & Rigaku, 2000 ▶); program(s) used to solve structure: *SHELXS97* (Sheldrick, 2008 ▶); program(s) used to refine structure: *SHELXL97* (Sheldrick, 2008 ▶); molecular graphics: *SHELXTL* (Sheldrick, 2008 ▶); software used to prepare material for publication: *SHELXTL*.

## Supplementary Material

Crystal structure: contains datablocks I, global. DOI: 10.1107/S1600536810017794/hg2677sup1.cif

Structure factors: contains datablocks I. DOI: 10.1107/S1600536810017794/hg2677Isup2.hkl

Additional supplementary materials:  crystallographic information; 3D view; checkCIF report

## Figures and Tables

**Table 1:** Hydrogen-bond geometry (Å, °)

*D*—H⋯*A*	*D*—H	H⋯*A*	*D*⋯*A*	*D*—H⋯*A*
N8—H8*B*⋯O2^i^	0.87 (6)	2.17 (6)	3.011 (6)	163 (5)
N4—H4*A*⋯N3^ii^	0.82 (5)	2.23 (5)	3.003 (6)	156 (5)
C12—H12⋯O1^iii^	0.95	2.32	3.248 (6)	165

Symmetry codes: (i) 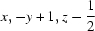; (ii) 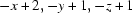; (iii) 
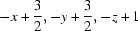
.

**Fig. 1.**
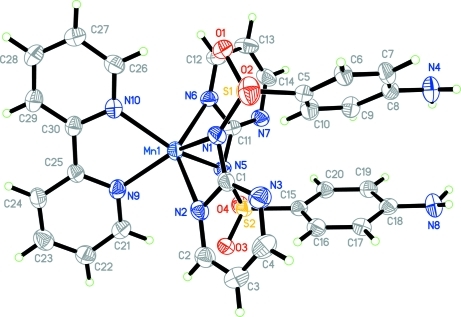
The molecular structure, showing the atom-labelling scheme. Displacement ellipsoids are drawn at the 50% probability level.

**Fig. 2.**
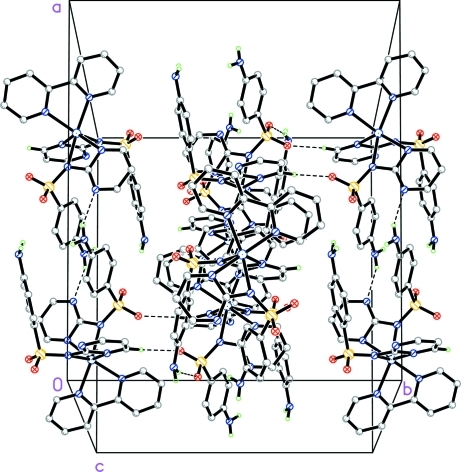
A packing plot of the title compound view along [001]. Hydrogen bonds are shown in the dashed line, and the H atoms that are not involved in hydrogen bonding are omitted.

## supplementary crystallographic information

### Comment

In the title compound, [Mn(sdz)_2_(bpy)] (where sdz is the sulfadiazine anion and bpy is 2,2'-bipyridine), the Mn(II) ion has six-coordinated distorted octahedral geometry and contains two bidentate N-coordinated sulfadiazinate anions and one chelating 2,2'-bipyridine ligand. The coordination mode of sulfadiazine is similar to its cobalt(II) complex (Ajibade *et al.*, 2006; Wang *et al.* 2010), nickel(II) complex (Wang *et al.*200 9), and copper(II) complex (Brown *et al.*, 1987), but different from Zn(sdz)_2_ (Yuan *et al.*, 2001), polymeric Cd(II) complex (Wang *et al.* 2005), and its copper complex (Hossain *et al.* 2006). The Mn—N bond distances involving the sulfonamide atoms N1, N5,the pyrimido atoms N2, N6, and the 2,2'-bipyridine atoms N9, N10, are very similar, at 2.242 (4), 2.234 (4), 2.312 (4), 2.278 (4), 2.235 (4), 2.225 (4) Å, respectively. The bond distances of the chelating bpy to Mn(II), Mn—N are consistent with those for the reported bpy-Mn(II) complexes, e.g. diazidobis(2,2'-bipyridine)manganese(II) (Mn—N 2.322 (3) Å)(Cheng *et al.*, 2004) and tris(2,2'-bipyridine)manganese(II) perchlorate hemihydrate (Mn—N 2.214 (4)-2.294 (4) Å)(Chen *et al.*, 1995). The tetrahedral coordination at S is distorted, also found in the neutral sulfadiazine molecule. A three dimensional network is generated via N—H···N, N—H···O, C—H···O hydrogen bonds from the sulfadiazine ligands of the complex.

### Experimental

0.1 mmol Mn(CH_3_COO)_2_.4H_2_O, 0.2 mmol sulfadiazine, 0.1 mmol 2,2'-bipyridine, ethanol (1 ml), methanol (1 ml) and pyridine (0.1 ml) were placed in a Pyrex tube (ca 25 cm). The tube was frozen with liquid N_2_, evacuated under vacuum, sealed with a torch and heated at 353 K for three days to give yellow block-shaped crystals, with a yield of 70%.

### Refinement

The carbon H atoms were treated as riding, with C—H distances of 0.95 Å ,and U_iso_(H) = 1.2U_eq_ (C). The H atoms attached to the amino N atoms were located in an electron-density map and refined isotropically.

### Crystal data

**Table:**

[Mn(C_10_H_9_N_4_O_2_S)_2_(C_10_H_8_N_2_)]	*F*(000) = 2920
*M**_r_* = 709.67	*D*_x_ = 1.554 Mg m^−^^3^
Monoclinic, *C*2/*c*	Mo *K*α radiation, λ = 0.71070 Å
Hall symbol: -C 2yc	Cell parameters from 8748 reflections
*a* = 20.121 (3) Å	θ = 3.1–25.3°
*b* = 17.555 (3) Å	µ = 0.63 mm^−^^1^
*c* = 17.956 (3) Å	*T* = 193 K
β = 106.973 (4)°	Block, yellow
*V* = 6066.4 (17) Å^3^	0.25 × 0.17 × 0.15 mm
*Z* = 8	

### Data collection

**Table:**

Rigaku Mercury CCD diffractometer	5533 independent reflections
Radiation source: fine-focus sealed tube	4438 reflections with *I* > 2σ(*I*)
graphite	*R*_int_ = 0.077
Detector resolution: 7.31 pixels mm^-1^	θ_max_ = 25.3°, θ_min_ = 3.1°
ω scans	*h* = −24→24
Absorption correction: multi-scan (REQAB; Jacobson, 1998)	*k* = −21→21
*T*_min_ = 0.859, *T*_max_ = 0.912	*l* = −19→21
28490 measured reflections	

### Refinement

**Table:**

Refinement on *F*^2^	Primary atom site location: structure-invariant direct methods
Least-squares matrix: full	Secondary atom site location: difference Fourier map
*R*[*F*^2^ > 2σ(*F*^2^)] = 0.073	Hydrogen site location: inferred from neighbouring sites
*wR*(*F*^2^) = 0.167	H atoms treated by a mixture of independent and constrained refinement
*S* = 1.17	*w* = 1/[σ^2^(*F*_o_^2^) + (0.0668*P*)^2^ + 12.6903*P*] where *P* = (*F*_o_^2^ + 2*F*_c_^2^)/3
5533 reflections	(Δ/σ)_max_ < 0.001
441 parameters	Δρ_max_ = 0.43 e Å^−^^3^
0 restraints	Δρ_min_ = −0.58 e Å^−^^3^

### Special details

**Table:**

Geometry. All esds (except the esd in the dihedral angle between two l.s. planes) are estimated using the full covariance matrix. The cell esds are taken into account individually in the estimation of esds in distances, angles and torsion angles; correlations between esds in cell parameters are only used when they are defined by crystal symmetry. An approximate (isotropic) treatment of cell esds is used for estimating esds involving l.s. planes.
Refinement. Refinement of *F*^2^ against ALL reflections. The weighted *R*-factor wR and goodness of fit *S* are based on *F*^2^, conventional *R*-factors *R* are based on *F*, with *F* set to zero for negative *F*^2^. The threshold expression of *F*^2^ > σ(*F*^2^) is used only for calculating *R*-factors(gt) etc. and is not relevant to the choice of reflections for refinement. *R*-factors based on *F*^2^ are statistically about twice as large as those based on *F*, and *R*- factors based on ALL data will be even larger.

### Fractional atomic coordinates and isotropic or equivalent isotropic displacement parameters (Å^2^)

**Table:**

	*x*	*y*	*z*	*U*_iso_*/*U*_eq_	
Mn1	0.68247 (3)	0.52337 (4)	0.53748 (4)	0.0286 (2)	
S1	0.85240 (6)	0.60913 (7)	0.57746 (7)	0.0328 (3)	
S2	0.66371 (6)	0.37074 (6)	0.39626 (7)	0.0293 (3)	
O1	0.81279 (17)	0.67735 (18)	0.5490 (2)	0.0428 (9)	
O2	0.90959 (17)	0.6182 (2)	0.6479 (2)	0.0441 (9)	
O3	0.66571 (17)	0.32382 (17)	0.46238 (18)	0.0364 (8)	
O4	0.60548 (15)	0.36038 (18)	0.32761 (19)	0.0355 (8)	
N1	0.79647 (18)	0.5472 (2)	0.5858 (2)	0.0303 (9)	
N2	0.75742 (18)	0.4341 (2)	0.6121 (2)	0.0304 (9)	
N3	0.8801 (2)	0.4501 (2)	0.6411 (2)	0.0386 (10)	
N4	0.9673 (3)	0.5174 (3)	0.3230 (3)	0.0417 (11)	
N5	0.66857 (19)	0.45691 (19)	0.4279 (2)	0.0280 (8)	
N6	0.67347 (17)	0.5838 (2)	0.4227 (2)	0.0284 (8)	
N7	0.6663 (2)	0.5141 (2)	0.3060 (2)	0.0327 (9)	
N8	0.9195 (2)	0.3105 (3)	0.3033 (3)	0.0414 (11)	
N9	0.59065 (18)	0.4786 (2)	0.5698 (2)	0.0316 (9)	
N10	0.63904 (19)	0.6209 (2)	0.5872 (2)	0.0315 (9)	
C1	0.8146 (2)	0.4764 (2)	0.6150 (3)	0.0283 (10)	
C2	0.7671 (3)	0.3623 (3)	0.6383 (3)	0.0374 (11)	
H2	0.7279	0.3313	0.6362	0.045*	
C3	0.8325 (3)	0.3328 (3)	0.6680 (3)	0.0422 (12)	
H3	0.8395	0.2824	0.6881	0.051*	
C4	0.8875 (3)	0.3790 (3)	0.6675 (3)	0.0432 (13)	
H4	0.9332	0.3590	0.6870	0.052*	
C5	0.8884 (2)	0.5770 (2)	0.5047 (3)	0.0290 (10)	
C6	0.9558 (2)	0.5958 (3)	0.5071 (3)	0.0333 (11)	
H6	0.9845	0.6221	0.5510	0.040*	
C7	0.9814 (2)	0.5768 (3)	0.4471 (3)	0.0344 (11)	
H7	1.0277	0.5906	0.4496	0.041*	
C8	0.9409 (2)	0.5373 (3)	0.3821 (3)	0.0314 (10)	
C9	0.8722 (2)	0.5200 (3)	0.3791 (3)	0.0344 (11)	
H9	0.8429	0.4950	0.3347	0.041*	
C10	0.8469 (2)	0.5389 (3)	0.4396 (3)	0.0378 (12)	
H10	0.8005	0.5258	0.4372	0.045*	
C11	0.6687 (2)	0.5180 (2)	0.3812 (3)	0.0280 (10)	
C12	0.6778 (2)	0.6488 (3)	0.3868 (3)	0.0388 (12)	
H12	0.6813	0.6955	0.4144	0.047*	
C13	0.6774 (3)	0.6497 (3)	0.3098 (3)	0.0453 (13)	
H13	0.6811	0.6960	0.2839	0.054*	
C14	0.6713 (3)	0.5814 (3)	0.2727 (3)	0.0401 (12)	
H14	0.6707	0.5814	0.2195	0.048*	
C15	0.7400 (2)	0.3524 (2)	0.3695 (3)	0.0293 (10)	
C16	0.8007 (2)	0.3292 (3)	0.4249 (3)	0.0334 (11)	
H16	0.8012	0.3229	0.4777	0.040*	
C17	0.8598 (2)	0.3153 (2)	0.4036 (3)	0.0308 (10)	
H17	0.9009	0.2994	0.4418	0.037*	
C18	0.8604 (2)	0.3243 (2)	0.3260 (3)	0.0311 (10)	
C19	0.7986 (2)	0.3457 (2)	0.2706 (3)	0.0324 (10)	
H19	0.7976	0.3505	0.2176	0.039*	
C20	0.7388 (2)	0.3599 (2)	0.2918 (3)	0.0310 (10)	
H20	0.6972	0.3747	0.2536	0.037*	
C21	0.5717 (3)	0.4048 (3)	0.5639 (3)	0.0402 (12)	
H21	0.5916	0.3714	0.5347	0.048*	
C22	0.5244 (3)	0.3759 (3)	0.5988 (3)	0.0426 (12)	
H22	0.5121	0.3235	0.5935	0.051*	
C23	0.4952 (3)	0.4237 (3)	0.6412 (3)	0.0426 (12)	
H23	0.4634	0.4045	0.6667	0.051*	
C24	0.5127 (2)	0.5001 (3)	0.6465 (3)	0.0350 (11)	
H24	0.4924	0.5344	0.6746	0.042*	
C25	0.5604 (2)	0.5258 (3)	0.6099 (3)	0.0292 (10)	
C26	0.6618 (2)	0.6924 (3)	0.5882 (3)	0.0348 (11)	
H26	0.7013	0.7021	0.5708	0.042*	
C27	0.6300 (3)	0.7526 (3)	0.6135 (3)	0.0398 (12)	
H27	0.6475	0.8029	0.6133	0.048*	
C28	0.5731 (3)	0.7392 (3)	0.6389 (3)	0.0425 (12)	
H28	0.5506	0.7800	0.6566	0.051*	
C29	0.5491 (2)	0.6656 (3)	0.6382 (3)	0.0381 (12)	
H29	0.5096	0.6552	0.6554	0.046*	
C30	0.5827 (2)	0.6070 (3)	0.6125 (3)	0.0304 (10)	
H4A	1.009 (3)	0.512 (3)	0.330 (3)	0.039 (15)*	
H4B	0.943 (3)	0.485 (3)	0.286 (3)	0.042 (15)*	
H8A	0.962 (3)	0.319 (3)	0.336 (4)	0.065 (19)*	
H8B	0.922 (3)	0.323 (3)	0.258 (3)	0.051 (17)*	

### Atomic displacement parameters (Å^2^)

**Table:**

	*U*^11^	*U*^22^	*U*^33^	*U*^12^	*U*^13^	*U*^23^
Mn1	0.0292 (4)	0.0308 (4)	0.0283 (4)	0.0005 (3)	0.0123 (3)	−0.0015 (3)
S1	0.0325 (6)	0.0312 (6)	0.0383 (7)	−0.0039 (5)	0.0159 (5)	−0.0080 (5)
S2	0.0351 (6)	0.0248 (6)	0.0311 (6)	−0.0019 (5)	0.0143 (5)	−0.0008 (5)
O1	0.0468 (19)	0.0261 (17)	0.065 (2)	−0.0001 (15)	0.0307 (18)	−0.0028 (16)
O2	0.0404 (19)	0.054 (2)	0.039 (2)	−0.0082 (16)	0.0144 (16)	−0.0165 (16)
O3	0.0485 (19)	0.0284 (17)	0.0398 (19)	0.0005 (15)	0.0247 (16)	0.0037 (14)
O4	0.0298 (16)	0.0360 (18)	0.0414 (19)	−0.0066 (14)	0.0114 (15)	−0.0060 (15)
N1	0.0296 (19)	0.029 (2)	0.035 (2)	−0.0042 (16)	0.0128 (17)	−0.0001 (16)
N2	0.0296 (19)	0.031 (2)	0.032 (2)	−0.0076 (17)	0.0123 (17)	−0.0053 (16)
N3	0.034 (2)	0.037 (2)	0.043 (2)	0.0035 (19)	0.0091 (19)	0.0048 (19)
N4	0.033 (2)	0.060 (3)	0.035 (3)	0.003 (2)	0.015 (2)	−0.005 (2)
N5	0.037 (2)	0.0215 (19)	0.026 (2)	0.0002 (16)	0.0103 (17)	−0.0018 (15)
N6	0.0255 (19)	0.0230 (19)	0.036 (2)	−0.0010 (15)	0.0078 (17)	0.0000 (16)
N7	0.040 (2)	0.032 (2)	0.028 (2)	−0.0010 (18)	0.0122 (18)	0.0025 (16)
N8	0.035 (2)	0.053 (3)	0.040 (3)	−0.003 (2)	0.017 (2)	−0.005 (2)
N9	0.0280 (19)	0.037 (2)	0.030 (2)	0.0054 (17)	0.0094 (17)	0.0017 (17)
N10	0.031 (2)	0.034 (2)	0.031 (2)	0.0008 (17)	0.0123 (17)	−0.0013 (16)
C1	0.031 (2)	0.030 (2)	0.027 (2)	−0.003 (2)	0.014 (2)	−0.0031 (19)
C2	0.046 (3)	0.036 (3)	0.033 (3)	−0.004 (2)	0.016 (2)	−0.003 (2)
C3	0.046 (3)	0.034 (3)	0.044 (3)	0.005 (2)	0.008 (2)	0.001 (2)
C4	0.037 (3)	0.043 (3)	0.044 (3)	0.008 (2)	0.003 (2)	0.000 (2)
C5	0.030 (2)	0.025 (2)	0.033 (3)	−0.0008 (19)	0.012 (2)	0.0013 (19)
C6	0.030 (2)	0.038 (3)	0.034 (3)	−0.004 (2)	0.013 (2)	−0.002 (2)
C7	0.025 (2)	0.043 (3)	0.038 (3)	−0.002 (2)	0.013 (2)	0.002 (2)
C8	0.036 (3)	0.031 (2)	0.031 (3)	0.005 (2)	0.015 (2)	0.0061 (19)
C9	0.030 (2)	0.038 (3)	0.034 (3)	−0.002 (2)	0.008 (2)	−0.007 (2)
C10	0.034 (3)	0.040 (3)	0.042 (3)	−0.004 (2)	0.015 (2)	−0.008 (2)
C11	0.031 (2)	0.022 (2)	0.029 (2)	−0.0001 (19)	0.007 (2)	0.0000 (18)
C12	0.043 (3)	0.025 (3)	0.049 (3)	−0.002 (2)	0.014 (2)	0.003 (2)
C13	0.060 (3)	0.032 (3)	0.042 (3)	−0.004 (3)	0.013 (3)	0.011 (2)
C14	0.050 (3)	0.040 (3)	0.033 (3)	−0.005 (2)	0.016 (2)	0.008 (2)
C15	0.036 (2)	0.023 (2)	0.030 (2)	−0.001 (2)	0.012 (2)	−0.0006 (18)
C16	0.042 (3)	0.029 (2)	0.030 (3)	−0.003 (2)	0.013 (2)	−0.004 (2)
C17	0.031 (2)	0.031 (2)	0.029 (2)	−0.003 (2)	0.006 (2)	0.0013 (19)
C18	0.037 (3)	0.023 (2)	0.033 (3)	−0.003 (2)	0.011 (2)	−0.0049 (19)
C19	0.045 (3)	0.030 (2)	0.025 (2)	−0.001 (2)	0.014 (2)	−0.0019 (19)
C20	0.034 (2)	0.030 (2)	0.029 (3)	−0.001 (2)	0.009 (2)	0.0005 (19)
C21	0.044 (3)	0.031 (3)	0.049 (3)	0.003 (2)	0.019 (3)	−0.002 (2)
C22	0.043 (3)	0.035 (3)	0.054 (3)	−0.004 (2)	0.020 (3)	0.001 (2)
C23	0.034 (3)	0.050 (3)	0.044 (3)	0.001 (2)	0.013 (2)	0.009 (2)
C24	0.032 (2)	0.044 (3)	0.031 (3)	0.007 (2)	0.014 (2)	0.005 (2)
C25	0.026 (2)	0.034 (3)	0.028 (2)	0.0003 (19)	0.008 (2)	0.0029 (19)
C26	0.038 (3)	0.034 (3)	0.033 (3)	−0.006 (2)	0.011 (2)	0.001 (2)
C27	0.045 (3)	0.029 (3)	0.048 (3)	−0.001 (2)	0.017 (3)	−0.001 (2)
C28	0.044 (3)	0.034 (3)	0.053 (3)	0.011 (2)	0.020 (3)	−0.001 (2)
C29	0.032 (2)	0.044 (3)	0.039 (3)	0.007 (2)	0.013 (2)	−0.003 (2)
C30	0.029 (2)	0.036 (3)	0.025 (2)	0.004 (2)	0.0068 (19)	−0.0015 (19)

### Geometric parameters (Å, °)

**Table:**

Mn1—N10	2.225 (4)	C6—C7	1.365 (6)
Mn1—N5	2.234 (4)	C6—H6	0.9500
Mn1—N9	2.235 (4)	C7—C8	1.396 (6)
Mn1—N1	2.242 (4)	C7—H7	0.9500
Mn1—N6	2.278 (4)	C8—C9	1.401 (6)
Mn1—N2	2.312 (4)	C9—C10	1.367 (7)
S1—O1	1.447 (4)	C9—H9	0.9500
S1—O2	1.449 (4)	C10—H10	0.9500
S1—N1	1.603 (4)	C12—C13	1.379 (7)
S1—C5	1.761 (4)	C12—H12	0.9500
S2—O3	1.436 (3)	C13—C14	1.360 (7)
S2—O4	1.443 (3)	C13—H13	0.9500
S2—N5	1.609 (4)	C14—H14	0.9500
S2—C15	1.766 (4)	C15—C16	1.393 (6)
N1—C1	1.356 (6)	C15—C20	1.395 (6)
N2—C2	1.339 (6)	C16—C17	1.374 (6)
N2—C1	1.359 (5)	C16—H16	0.9500
N3—C4	1.328 (6)	C17—C18	1.405 (6)
N3—C1	1.344 (6)	C17—H17	0.9500
N4—C8	1.364 (6)	C18—C19	1.397 (6)
N4—H4A	0.82 (5)	C19—C20	1.388 (6)
N4—H4B	0.89 (6)	C19—H19	0.9500
N5—C11	1.361 (5)	C20—H20	0.9500
N6—C12	1.326 (6)	C21—C22	1.380 (7)
N6—C11	1.363 (5)	C21—H21	0.9500
N7—C11	1.340 (6)	C22—C23	1.376 (7)
N7—C14	1.341 (6)	C22—H22	0.9500
N8—C18	1.387 (6)	C23—C24	1.383 (7)
N8—H8A	0.91 (6)	C23—H23	0.9500
N8—H8B	0.87 (6)	C24—C25	1.387 (6)
N9—C21	1.345 (6)	C24—H24	0.9500
N9—C25	1.354 (6)	C25—C30	1.492 (6)
N10—C26	1.333 (6)	C26—C27	1.379 (6)
N10—C30	1.360 (6)	C26—H26	0.9500
C2—C3	1.369 (7)	C27—C28	1.372 (7)
C2—H2	0.9500	C27—H27	0.9500
C3—C4	1.375 (7)	C28—C29	1.379 (7)
C3—H3	0.9500	C28—H28	0.9500
C4—H4	0.9500	C29—C30	1.383 (6)
C5—C6	1.383 (6)	C29—H29	0.9500
C5—C10	1.395 (6)		
			
N10—Mn1—N5	142.44 (13)	C6—C7—H7	119.4
N10—Mn1—N9	73.45 (14)	C8—C7—H7	119.4
N5—Mn1—N9	98.33 (14)	N4—C8—C7	120.8 (4)
N10—Mn1—N1	100.29 (14)	N4—C8—C9	121.2 (5)
N5—Mn1—N1	106.80 (13)	C7—C8—C9	118.0 (4)
N9—Mn1—N1	142.64 (14)	C10—C9—C8	120.6 (4)
N10—Mn1—N6	93.88 (13)	C10—C9—H9	119.7
N5—Mn1—N6	59.32 (13)	C8—C9—H9	119.7
N9—Mn1—N6	123.06 (13)	C9—C10—C5	120.7 (4)
N1—Mn1—N6	93.76 (13)	C9—C10—H10	119.6
N10—Mn1—N2	123.73 (13)	C5—C10—H10	119.6
N5—Mn1—N2	92.83 (13)	N7—C11—N5	125.1 (4)
N9—Mn1—N2	93.42 (13)	N7—C11—N6	124.8 (4)
N1—Mn1—N2	58.77 (13)	N5—C11—N6	110.1 (4)
N6—Mn1—N2	134.94 (13)	N6—C12—C13	121.0 (5)
O1—S1—O2	115.6 (2)	N6—C12—H12	119.5
O1—S1—N1	105.4 (2)	C13—C12—H12	119.5
O2—S1—N1	113.2 (2)	C14—C13—C12	117.1 (4)
O1—S1—C5	107.9 (2)	C14—C13—H13	121.4
O2—S1—C5	106.8 (2)	C12—C13—H13	121.4
N1—S1—C5	107.7 (2)	N7—C14—C13	124.4 (5)
O3—S2—O4	117.0 (2)	N7—C14—H14	117.8
O3—S2—N5	105.28 (19)	C13—C14—H14	117.8
O4—S2—N5	111.74 (19)	C16—C15—C20	119.9 (4)
O3—S2—C15	107.7 (2)	C16—C15—S2	120.7 (3)
O4—S2—C15	107.3 (2)	C20—C15—S2	119.5 (3)
N5—S2—C15	107.4 (2)	C17—C16—C15	120.2 (4)
C1—N1—S1	122.7 (3)	C17—C16—H16	119.9
C1—N1—Mn1	96.3 (3)	C15—C16—H16	119.9
S1—N1—Mn1	139.2 (2)	C16—C17—C18	121.0 (4)
C2—N2—C1	117.8 (4)	C16—C17—H17	119.5
C2—N2—Mn1	147.7 (3)	C18—C17—H17	119.5
C1—N2—Mn1	93.1 (3)	N8—C18—C19	119.9 (4)
C4—N3—C1	116.4 (4)	N8—C18—C17	121.9 (4)
C8—N4—H4A	122 (4)	C19—C18—C17	118.2 (4)
C8—N4—H4B	119 (3)	C20—C19—C18	121.1 (4)
H4A—N4—H4B	111 (5)	C20—C19—H19	119.4
C11—N5—S2	122.3 (3)	C18—C19—H19	119.4
C11—N5—Mn1	96.2 (3)	C19—C20—C15	119.6 (4)
S2—N5—Mn1	141.3 (2)	C19—C20—H20	120.2
C12—N6—C11	117.9 (4)	C15—C20—H20	120.2
C12—N6—Mn1	147.3 (3)	N9—C21—C22	122.4 (4)
C11—N6—Mn1	94.2 (3)	N9—C21—H21	118.8
C11—N7—C14	114.8 (4)	C22—C21—H21	118.8
C18—N8—H8A	121 (4)	C23—C22—C21	119.3 (5)
C18—N8—H8B	122 (4)	C23—C22—H22	120.4
H8A—N8—H8B	106 (5)	C21—C22—H22	120.4
C21—N9—C25	118.0 (4)	C22—C23—C24	119.2 (5)
C21—N9—Mn1	123.7 (3)	C22—C23—H23	120.4
C25—N9—Mn1	117.2 (3)	C24—C23—H23	120.4
C26—N10—C30	118.8 (4)	C23—C24—C25	118.7 (4)
C26—N10—Mn1	123.8 (3)	C23—C24—H24	120.6
C30—N10—Mn1	117.3 (3)	C25—C24—H24	120.6
N3—C1—N1	125.1 (4)	N9—C25—C24	122.3 (4)
N3—C1—N2	124.0 (4)	N9—C25—C30	115.0 (4)
N1—C1—N2	110.9 (4)	C24—C25—C30	122.7 (4)
N2—C2—C3	121.2 (5)	N10—C26—C27	122.4 (4)
N2—C2—H2	119.4	N10—C26—H26	118.8
C3—C2—H2	119.4	C27—C26—H26	118.8
C2—C3—C4	117.3 (5)	C28—C27—C26	119.3 (5)
C2—C3—H3	121.4	C28—C27—H27	120.3
C4—C3—H3	121.4	C26—C27—H27	120.3
N3—C4—C3	123.3 (5)	C27—C28—C29	118.9 (5)
N3—C4—H4	118.3	C27—C28—H28	120.6
C3—C4—H4	118.3	C29—C28—H28	120.6
C6—C5—C10	118.8 (4)	C28—C29—C30	119.7 (4)
C6—C5—S1	120.9 (3)	C28—C29—H29	120.2
C10—C5—S1	120.0 (3)	C30—C29—H29	120.2
C7—C6—C5	120.7 (4)	N10—C30—C29	121.0 (4)
C7—C6—H6	119.7	N10—C30—C25	115.8 (4)
C5—C6—H6	119.7	C29—C30—C25	123.3 (4)
C6—C7—C8	121.1 (4)		
			
O1—S1—N1—C1	−176.6 (3)	C2—N2—C1—N1	179.3 (4)
O2—S1—N1—C1	−49.3 (4)	Mn1—N2—C1—N1	9.4 (4)
C5—S1—N1—C1	68.5 (4)	C1—N2—C2—C3	0.9 (7)
O1—S1—N1—Mn1	23.2 (4)	Mn1—N2—C2—C3	161.8 (4)
O2—S1—N1—Mn1	150.4 (3)	N2—C2—C3—C4	−2.0 (7)
C5—S1—N1—Mn1	−91.8 (3)	C1—N3—C4—C3	0.8 (7)
N10—Mn1—N1—C1	129.6 (3)	C2—C3—C4—N3	1.1 (8)
N5—Mn1—N1—C1	−76.7 (3)	O1—S1—C5—C6	96.4 (4)
N9—Mn1—N1—C1	53.5 (4)	O2—S1—C5—C6	−28.5 (4)
N6—Mn1—N1—C1	−135.7 (3)	N1—S1—C5—C6	−150.3 (4)
N2—Mn1—N1—C1	6.3 (2)	O1—S1—C5—C10	−77.0 (4)
N10—Mn1—N1—S1	−67.0 (3)	O2—S1—C5—C10	158.2 (4)
N5—Mn1—N1—S1	86.7 (3)	N1—S1—C5—C10	36.3 (4)
N9—Mn1—N1—S1	−143.2 (3)	C10—C5—C6—C7	−0.5 (7)
N6—Mn1—N1—S1	27.6 (3)	S1—C5—C6—C7	−174.0 (4)
N2—Mn1—N1—S1	169.6 (4)	C5—C6—C7—C8	−0.6 (7)
N10—Mn1—N2—C2	109.5 (6)	C6—C7—C8—N4	−178.9 (5)
N5—Mn1—N2—C2	−61.4 (6)	C6—C7—C8—C9	2.1 (7)
N9—Mn1—N2—C2	37.1 (6)	N4—C8—C9—C10	178.5 (5)
N1—Mn1—N2—C2	−169.4 (6)	C7—C8—C9—C10	−2.4 (7)
N6—Mn1—N2—C2	−109.1 (6)	C8—C9—C10—C5	1.3 (7)
N10—Mn1—N2—C1	−87.4 (3)	C6—C5—C10—C9	0.2 (7)
N5—Mn1—N2—C1	101.8 (3)	S1—C5—C10—C9	173.7 (4)
N9—Mn1—N2—C1	−159.7 (3)	C14—N7—C11—N5	−176.1 (4)
N1—Mn1—N2—C1	−6.2 (2)	C14—N7—C11—N6	2.2 (7)
N6—Mn1—N2—C1	54.0 (3)	S2—N5—C11—N7	−1.3 (6)
O3—S2—N5—C11	179.5 (3)	Mn1—N5—C11—N7	175.6 (4)
O4—S2—N5—C11	−52.6 (4)	S2—N5—C11—N6	−179.8 (3)
C15—S2—N5—C11	64.9 (4)	Mn1—N5—C11—N6	−2.9 (4)
O3—S2—N5—Mn1	4.3 (4)	C12—N6—C11—N7	−1.8 (6)
O4—S2—N5—Mn1	132.3 (3)	Mn1—N6—C11—N7	−175.7 (4)
C15—S2—N5—Mn1	−110.2 (3)	C12—N6—C11—N5	176.6 (4)
N10—Mn1—N5—C11	51.9 (4)	Mn1—N6—C11—N5	2.8 (4)
N9—Mn1—N5—C11	125.5 (3)	C11—N6—C12—C13	0.2 (7)
N1—Mn1—N5—C11	−82.4 (3)	Mn1—N6—C12—C13	168.7 (4)
N6—Mn1—N5—C11	1.9 (2)	N6—C12—C13—C14	0.8 (8)
N2—Mn1—N5—C11	−140.6 (3)	C11—N7—C14—C13	−1.0 (7)
N10—Mn1—N5—S2	−132.2 (3)	C12—C13—C14—N7	−0.4 (8)
N9—Mn1—N5—S2	−58.6 (3)	O3—S2—C15—C16	−28.5 (4)
N1—Mn1—N5—S2	93.4 (3)	O4—S2—C15—C16	−155.2 (4)
N6—Mn1—N5—S2	177.7 (4)	N5—S2—C15—C16	84.5 (4)
N2—Mn1—N5—S2	35.3 (3)	O3—S2—C15—C20	150.3 (3)
N10—Mn1—N6—C12	36.2 (6)	O4—S2—C15—C20	23.5 (4)
N5—Mn1—N6—C12	−171.7 (6)	N5—S2—C15—C20	−96.8 (4)
N9—Mn1—N6—C12	108.9 (6)	C20—C15—C16—C17	1.5 (7)
N1—Mn1—N6—C12	−64.4 (6)	S2—C15—C16—C17	−179.8 (3)
N2—Mn1—N6—C12	−112.5 (6)	C15—C16—C17—C18	0.1 (7)
N10—Mn1—N6—C11	−154.0 (3)	C16—C17—C18—N8	−179.9 (4)
N5—Mn1—N6—C11	−1.9 (2)	C16—C17—C18—C19	−1.7 (6)
N9—Mn1—N6—C11	−81.2 (3)	N8—C18—C19—C20	−179.9 (4)
N1—Mn1—N6—C11	105.5 (3)	C17—C18—C19—C20	1.9 (6)
N2—Mn1—N6—C11	57.4 (3)	C18—C19—C20—C15	−0.4 (7)
N10—Mn1—N9—C21	−175.6 (4)	C16—C15—C20—C19	−1.3 (6)
N5—Mn1—N9—C21	42.0 (4)	S2—C15—C20—C19	179.9 (3)
N1—Mn1—N9—C21	−90.3 (4)	C25—N9—C21—C22	−1.8 (7)
N6—Mn1—N9—C21	100.6 (4)	Mn1—N9—C21—C22	166.2 (4)
N2—Mn1—N9—C21	−51.4 (4)	N9—C21—C22—C23	0.0 (8)
N10—Mn1—N9—C25	−7.6 (3)	C21—C22—C23—C24	1.6 (8)
N5—Mn1—N9—C25	−150.0 (3)	C22—C23—C24—C25	−1.3 (7)
N1—Mn1—N9—C25	77.7 (4)	C21—N9—C25—C24	2.0 (6)
N6—Mn1—N9—C25	−91.3 (3)	Mn1—N9—C25—C24	−166.8 (3)
N2—Mn1—N9—C25	116.7 (3)	C21—N9—C25—C30	−178.7 (4)
N5—Mn1—N10—C26	−91.8 (4)	Mn1—N9—C25—C30	12.5 (5)
N9—Mn1—N10—C26	−173.9 (4)	C23—C24—C25—N9	−0.5 (7)
N1—Mn1—N10—C26	44.1 (4)	C23—C24—C25—C30	−179.7 (4)
N6—Mn1—N10—C26	−50.5 (4)	C30—N10—C26—C27	−0.3 (7)
N2—Mn1—N10—C26	103.2 (4)	Mn1—N10—C26—C27	174.4 (4)
N5—Mn1—N10—C30	83.0 (4)	N10—C26—C27—C28	0.2 (7)
N9—Mn1—N10—C30	1.0 (3)	C26—C27—C28—C29	−0.2 (8)
N1—Mn1—N10—C30	−141.1 (3)	C27—C28—C29—C30	0.3 (8)
N6—Mn1—N10—C30	124.4 (3)	C26—N10—C30—C29	0.5 (6)
N2—Mn1—N10—C30	−81.9 (3)	Mn1—N10—C30—C29	−174.6 (3)
C4—N3—C1—N1	−179.9 (4)	C26—N10—C30—C25	−179.9 (4)
C4—N3—C1—N2	−2.1 (7)	Mn1—N10—C30—C25	5.0 (5)
S1—N1—C1—N3	1.1 (6)	C28—C29—C30—N10	−0.4 (7)
Mn1—N1—C1—N3	168.3 (4)	C28—C29—C30—C25	179.9 (5)
S1—N1—C1—N2	−176.9 (3)	N9—C25—C30—N10	−11.6 (6)
Mn1—N1—C1—N2	−9.8 (4)	C24—C25—C30—N10	167.7 (4)
C2—N2—C1—N3	1.3 (7)	N9—C25—C30—C29	168.1 (4)
Mn1—N2—C1—N3	−168.6 (4)	C24—C25—C30—C29	−12.6 (7)

### Hydrogen-bond geometry (Å, °)

**Table:**

*D*—H···*A*	*D*—H	H···*A*	*D*···*A*	*D*—H···*A*
N8—H8B···O2^i^	0.87 (6)	2.17 (6)	3.011 (6)	163 (5)
N4—H4A···N3^ii^	0.82 (5)	2.23 (5)	3.003 (6)	156 (5)
C12—H12···O1^iii^	0.95	2.32	3.248 (6)	165

Symmetry codes: (i) *x*, −*y*+1, *z*−1/2; (ii) −*x*+2, −*y*+1, −*z*+1; (iii) −*x*+3/2, −*y*+3/2, −*z*+1.
